# Advocacy, Hesitancy, and Equity: Exploring U.S. Race-Related Discussions of the COVID-19 Vaccine on Twitter

**DOI:** 10.3390/ijerph18115693

**Published:** 2021-05-26

**Authors:** Shaniece Criss, Thu T. Nguyen, Samantha Norton, Imaya Virani, Eli Titherington, Emma Lou Tillmanns, Courtney Kinnane, Gabrielle Maiolo, Anne B. Kirby, Gilbert C. Gee

**Affiliations:** 1 Department of Health Sciences, Furman University, Greenville, SC 29613, USA; samantha.norton@furman.edu (S.N.); imaya.virani@furman.edu (I.V.); eli.titherington@furman.edu (E.T.); emma.tillmanns@furman.edu (E.L.T.); courtney.kinnane@furman.edu (C.K.); gabrielle.maiolo@furman.edu (G.M.); anne.kirby@furman.edu (A.B.K.); 2 Department of Family and Community Medicine, University of California, San Francisco, CA 94110, USA; thu.nguyen@ucsf.edu; 3 Department of Community Health Sciences, University of California, Los Angeles, CA 90095, USA; gilgee@ucla.edu

**Keywords:** social media, Twitter, vaccine, vaccine hesitancy, content analysis, people of color

## Abstract

Background: Our study aimed to describe themes of tweets related to COVID-19 vaccines, race, and ethnicity to explore the context of the intersection of these topics on Twitter. Methods: We utilized Twitter’s Streaming Application Programming Interface (API) to collect a random 1% sample of publicly available tweets from October 2020 to January 2021. The study team conducted a qualitative content analysis from the full data set of 1110 tweets. Results: The tweets revealed vaccine support through vaccine affirmation, advocacy through reproach, a need for a vaccine, COVID-19 and racism, vaccine development and efficacy, racist vaccine humor, and news updates. Vaccine opposition was demonstrated through direct opposition, vaccine hesitancy, and adverse reactions. Conspiracy and misinformation included scientific misinformation, political misinformation, beliefs about immunity and protective behaviors, and race extermination conspiracy. Equity and access focused on overcoming history of medical racism, pointing out health disparities, and facilitators to vaccine access. Representation touted pride in development and role models, and politics discussed the role of politics in vaccines and international politics. Conclusion: Our analysis demonstrates that Twitter can provide nuances about multiple viewpoints on the vaccine related to race and ethnicity and can be beneficial in contributing to insights for public health messaging.

## 1. Introduction

From September to December 2020, a nationally represented survey in the United States (U.S.) found that respondents’ intentions to take the vaccine rose from 39.4% to 49.1%, and non-intent decreased from 38.1% to 32.1% [1]. Racial and ethnic numerical minorities reported having greater vaccine hesitancy than White people. Specifically, studies have consistently found that Black and Hispanic people report higher levels of vaccine hesitancy than White people in the U.S. [2,3,4]. In addition, Asian people and people reporting more than one race or other have higher levels of vaccine hesitancy than White people [4]. As of April 2021, 65% of White people, 11% of Hispanic people, 9% of Black people, 1% of Asian people, 1% of American Indian or Alaska Native, and <1% of Native Hawaiian reported receiving at least one dose of a COVID-19 vaccine among the 55% of data that had race and ethnicity reported [5]. It appears that vaccine hesitancy may be one factor impacting vaccine uptake.

A driving factor for vaccine hesitancy among specific racial and ethnic groups is the history of and current racism in the U.S. healthcare system [6,7], from Tuskegee to the present day [8]. In a nationally representative study of U.S. adults ages 50+, health care discrimination was associated with higher odds of elevated HbA1c and C-reactive protein, a marker of inflammation and a predictor of coronary heart disease [9]. Black Americans report significantly more experiences of racial discrimination in health care settings, leading to apprehension towards individual providers, as well as mistrust of the overall healthcare system [6]. Discrimination in the healthcare system could impact how people perceive trust in a vaccine. For instance, the Black community reports less vaccine trust and higher levels of perceived potential harm from the vaccine compared to other races in the U.S. [10,11,12]. Americans who get immunized for the flu yearly describe having a trust in public health and government authorities, as well as having trust in vaccine production and health care providers administering it [7]. Yet, vaccine hesitancy among African Americans is connected to an increasing concern about the government’s motives [7,12].

The politicization of the COVID-19 vaccine increased in 2020 [11], which could have exacerbated concerns. Analyzing the context of the time period in which the pandemic has unfolded is critical to understanding COVID-19 vaccine hesitancy. Since the beginning of the COVID-19 pandemic, the U.S. has also undergone a historic presidential election and a significant rise in racial tensions and social justice movements. At the beginning of the pandemic, some elected officials continuously used offensive terms linking the coronavirus to the Asian community believed to fuel violence against the Asian American community and sparking a public outcry against the use of the racist terms [13,14]. During the summer social justice protests of 2020, members of the federal government utilized divisive rhetoric, leading to a further disconnect between elected officials and members of the Black community [15]. The use of discriminatory language established a level of distrust in the government among communities of color, which has resulted in concerns and hesitancy about the safety and efficacy of the COVID-19 vaccine. Consequently, communities of color have cited the federal government as the least reliable source of information regarding COVID-19, and doctors and other public health officials as being the most reliable [15]. It is paramount to understand the power of social media to influence behaviors such as COVID-19 vaccine uptake during this pandemic.

As news progressed about the vaccine production, social media outlets were used as a space to voice opinions about efficacy, equity, ethics, representation, and conspiracies surrounding this process [16,17]. Even before the pandemic, the anti-vaccination community has used social media to influence health decisions with their opinions about vaccines, and COVID-19 vaccines have not been an exception [18,19,20]. Misinformation on social media has played a major role within the COVID-19 pandemic, as there are no standards of regulation regarding quality, accuracy, and availability of the information [21]. Vaccine hesitancy is highly influenced by misinformation propagated on social media [22]. In addition, Twitter can be used to spread racism. Emboldened anonymity along with echo chambers can fuel racist tweets [23]. In particular, there has been a rise in negative tweets referencing Asians with the emergence of COVID-19 pandemic with the apex in negative Asian sentiment occurring during the week of March 16, when President Trump used the phrase, “Chinese virus” [14,24]. The intersection of racism and the pandemic could have a similar impact on vaccine perception.

With the availability of Pfizer, Moderna, and Johnson & Johnson vaccines in the U.S., uptake is of the utmost concern for curbing the pandemic. When more individuals become vaccinated in a community, the virus is less likely to be transmitted due to herd immunity [25]. Dr. Fauci stated that 70% to 85% of the American population needs to have the COVID-19 vaccine to achieve herd immunity [26]. Twitter provides a natural laboratory for researchers to study perceptions of the vaccine in order to understand public sentiment within this complex social and medical milieu. Our study aims to describe themes of tweets related to COVID-19 vaccines, race, and ethnicity to explore the context of the intersection of these topics on Twitter using qualitative content analysis. This type of analysis has the potential to contribute to message development to support vaccine uptake.

## 2. Methods

A random 1% sample of publicly available tweets in the United States was collected from October 2020 to January 2021, using Twitter’s Streaming Application Programming Interface (API) that included key words from all the categories of race/ethnicity from a keyword list of 518 race-related terms, COVID-19 from 75 coronavirus-related keywords [14], and vaccine from 28 vaccine-related keywords (Appendix A). Terms for vaccines included variations of vaccine, vaccination, immune, immunization, vax, and anti-vax. The first COVID-19 vaccine was given on 14 December 2020 in the U.S., so the study timeline captures the anticipation of having a vaccine, as well as early dissemination. The study team conducted a qualitative content analysis from the full data set of 1110 tweets. This study was determined exempt by the University of California, San Francisco Institutional Review Board.

The study team developed the codebook based on a literature review and reviewing tweets from the sample. Specifically, the team had an extensive multi-session discussion about the first 335 tweets from the sample to solidify the codes and definitions in the codebook. The final codes were vaccine support, vaccine opposition, conspiracy and misinformation, equity and access, representation, and politics. Using this coding scheme, all the tweets were double coded by two study members independently. Eight members of the study team discussed all discrepancies in the coding and came to a consensus on the final code for each tweet to achieve complete inter-rater reliability. Utilizing thematic analysis, the team analyzed tweets within each code to identify themes [27], which are explained in the results section. With this extensive consensus building process, we sought to maintain data trustworthiness through utilizing multiple data analysts with different racial backgrounds and life experiences.

## 3. Results

From the 1110 tweets, themes emerged from the overarching categories of vaccine support, vaccine opposition, conspiracy and misinformation, equity and access, representation, and politics. Table 1 provides the detailed themes of each category along with illustrative tweets. In general, many of the race terms in our sample were used as a descriptor, and some of were in a derogatory manner. Of note, many of the terms associated with Asian people utilized anti-Asian rhetoric related to the coronavirus like “Chinese virus.”

### 3.1. Vaccine Support

This category represented 21% of the sample. There were six themes in this section. *Vaccine affirmation* focused on tweets reporting the excitement that Black and Latinx people were getting the vaccine, as well as some Twitter users viewing it as an act of “dismantling systemic racism.” *Advocacy through reproach* were tweets that expressed agitation (many times through expletives or insults) that were “calling out” perceived despicable behavior as a way to assert that people should get vaccinated. The metaphor of *a need for a vaccine for COVID-19 and racism* recognizes the interplay between the pandemic and racial tension and the deep desire to remedy both of the issues. Vaccine support was also shown through positive tweets about *vaccine development and efficacy*. There was a trend of *racist vaccine humor* through stating what people already did (e.g., like eating certain types of food), and then saying “don’t worry what’s in the covid vaccine.” This pattern was used many times without race terms as well. *News updates* supported vaccine support by providing accurate, and potentially inspiring updates about the vaccine within communities of color.

### 3.2. Vaccine Opposition

This category represented 12% of the sample. There were three themes in this section. *Direct opposition* were tweets that strongly asserted that they would never take the vaccine. Tweets displayed *vaccine hesitancy* through expressing reservations about the vaccine and saying that they wanted to see other groups get the vaccine first or gain understanding of connections to negative consequences. Some tweets focused solely on potential or rare *adverse reactions.*

### 3.3. Conspiracy and Misinformation

This category represented 15% of the sample. There were four themes in this section. *Scientific misinformation* were tweets that directly provided information counter to research about the vaccine. *Political misinformation* focused on perceived government intervention that was not true, such as the government using vaccines to insert microchips and governmental vaccine use for population control. Some tweets focused on *beliefs about immunity and protective behaviors*, which focused on the strength of the immune system over and above the protection of the vaccine. The *race extermination conspiracy* tweets posited that the vaccine was created to “kill off [people of color] POC.”

### 3.4. Equity and Access

This category represented 18% of the sample with three themes emerging. *Overcoming history of medical racism* references tweets that acknowledge the past, but urges Black people to still get vaccinated. Tweets also focused on *pointing out health disparities* related to COVID-19 based on pre-existing conditions, hospitalization, and work conditions. Twitter users shared information to promote *facilitators to vaccine access* through prominent speakers and organizations that are from and represent communities of color, as well as petitions to demand access. 

### 3.5. Representation

This category represented 13% of the sample with two themes. Tweets highlighted *pride in development* through celebrating that a Black women developed the vaccine. Twitter users also emphasized the importance of *role models* from Black and Latinx communities by publicly taking the vaccine or promoting vaccine uptake. Role models included organizational leaders, civil rights leaders, and celebrities.

### 3.6. Politics

This category represented 20% of the sample. Twitter users discussed the *role of politics in vaccines* through mentioning politicians and political parties concerning their stance on mask mandates, access to the vaccine, speed of the vaccine development, and the presidential transition. Tweets also mentioned *international politics*, and they were commenting on the COVID-19 impact and vaccine roll-out in other countries.

## 4. Discussion

This is the first study, to our knowledge, to conduct a qualitative content analysis on the intersection of COVID-19 vaccines, race, and ethnicity. Our analysis demonstrates that Twitter can provide nuances about multiple viewpoints on the vaccine related to race and ethnicity. The tweets revealed vaccine support through vaccine affirmation, advocacy through reproach, a need for a vaccine for COVID-19 and racism, vaccine development and efficacy, racist vaccine humor, and news updates. Vaccine opposition was demonstrated through direct opposition, vaccine hesitancy, and adverse reactions. Conspiracy and misinformation included scientific misinformation, political misinformation, beliefs about immunity and protective behaviors, and race extermination conspiracy. Equity and access focused on overcoming history of medical racism, pointing out health disparities, and facilitators to vaccine access. Representation touted pride in development and role models, and politics discussed the role of politics in vaccines and international politics. The information gleaned from this qualitative content analysis can be beneficial in contributing to insights for public health messaging.

Discussion of COVID-19 on Twitter can display sentiments of anticipation, anger, and fear [28]. Some people may oppose vaccinations because of mistrust of healthcare providers and big pharma, denialism (“rhetoric employed in order to give the appearance of legitimate debate where there is none, with the goal of rejecting an argument for which there is a consensus of expert opinion”) and social media’s role in providing confirmation bias [29], (p. 4484). Our research found that COVID-19 vaccines are susceptible to the some of those factors. To address it, tactics could include engaging people with opposition and/or hesitancy through discussion with healthcare professionals and having open forums on social media sites [29,30]. Furthermore, COVID-19 vaccine opponents were more likely to share unreliable information (34.5%) compared to vaccine proponents (11.3%) [17]. Our data also shows that the public’s perception of the COVID-19 virus and vaccination may not align with accurate information and can even promote racism, like the use of the term “Chinese virus.”

Discussions around perception of the COVID-19 vaccine and access to the COVID-19 vaccine are important to analyze so adjustments can be made in the delivery and approach to deploying the vaccine [31]. Our study found tweets that were explicitly related to equity and access. Research demonstrates that the COVID-19 pandemic has disproportionately impacted communities of color due to socioeconomic status and lack of access [32]. Moreover, older Americans living in low-income households who are Black or of American Indian race have a higher associated risk of illness from COVID-19 [31,33]. Since equitable COVID-19 distribution is linked to removing the associated barriers [34], Twitter sentiment under the theme of equity and access communicates a relevance for vaccine equity and a priority of vaccine coverage to go to individuals at a higher risk of infection [31,33]. Our study findings support the perception that equitable access to the COVID-19 vaccine rollout should be addressed amidst barriers by race and socioeconomic status.

Representation is an area that could be utilized for positive messaging about the vaccine. In our study, tweets referenced Dr. Kizzmekia Corbett’s work in the Moderna vaccine development process. In recent years, Twitter has been used by leaders and officials from all over the world to communicate information about policies and current events. This uptake by world leaders contributes to the growing influence of social media on behaviors of citizens, especially during times of crisis [35]. Willingness to receive the COVID-19 vaccine was associated with recommendations made by former Vice President Biden, the Centers for Disease Control and Prevention, or the World Health Organization [12]. There is a significant correlation between vaccine trust and an individual’s trust of news sources who promote trust in vaccines [11]. There is an opportunity to partner with leaders identified on Twitter to share information about the benefits of vaccine development. For instance, there could be a Twitter campaign that features Dr. Corbett, as well as other significant leaders in this area. Additionally, our study included exemplary messaging expressing vaccine support that could potentially be utilized, along with different approaches taken, including affirmations and attempted humor. In fact, research shows that humor helps exhibit positivity and a sense of cohesion during COVID-19 [36]. Yet, it is important that the humor does not include racist tones like the tweets in our study.

Many of the political tweets expressed conflict and disapproval. Previous research has suggested that the age of social media as a primary form of news distribution has created a space in which individuals can selectively expose themselves to information, furthering the divide between political parties and crafting one-sided narratives based on a predetermined set of political views [37]. Research on the varying sources of COVID-19 misinformation finds that misinformation issued by politicians garners more attention and a more significant reaction than misinformation from many other sources due to their high-profile nature [38]. Spread of this misinformation from politicians themselves as well as their supporters may be incentivized by political motives [38]. Overall, the current climate of political polarization makes social media a breeding ground for the spread of misinformation, political incentivizing, and selective distribution of facts regarding COVID-19 vaccines. A media approach could attempt to disentangle the science from the political process.

The approach to this content analysis study has some limitations. The process by which the tweets were acquired does not provide information about the users’ identity; therefore, the race or ethnic groups with which they identify were unclear. However, it is clear which race and ethnic groups were referred to in the tweets, so it provides information about how people are discussing these groups in public discourse. The API system does not collect the tweets within the context of a conversation or an algorithm for the user, so some meaning may have been unclear and individual exposure to tweets is undetermined. Yet, the number of tweets collected provided an overall sentiment of the main ideas. In addition, a team of eight people discussed all tweets with discrepancies in order to grapple with the interpretation. While the study used some previously published vaccine-related terms [39], it is possible that the keywords used for this data collection may not completely reflect the terminology used to discuss the topic, therefore not capturing all relevant tweets.

## 5. Conclusions

Due to the health disparities present in the United States related to race, it is vital to distribute the COVID-19 vaccine in communities of color. As seen in the results of this study, a segment of Twitter users expressed their hesitancy toward the vaccine. This was attributed to several causes as discussed, but we recommend the practice of utilizing qualitative analysis of Twitter data to potentially contribute to the development of messaging for public health campaigns. For instance, the analysis can help with message development, and then the messages would need to be tested with the intended audiences before dissemination. Our study showed how this type of analysis can offer nuanced insight about public sentiment, as well as areas to target in a media outreach, from who should be the messenger to the specific topic. Minimally, public health practitioners can encourage and amplify positive sentiments and affirmations of the vaccine on social media. A coordinated effort could potentially result in contributing to attitude change about vaccine uptake.

## Figures and Tables

**Table 1:** Content analysis themes of tweets related to COVID-19 vaccines and race/ethnicity with illustrative examples, from sample of 1110 tweets from October 2020 to January 2021.

Themes	Example Tweets
Vaccine support (229 tweets; 21% of the sample)	
Vaccine affirmation	Received my first dose of the COVID-19 vaccine yesterday and I could not be more excited! Shoutout to La Clinica Latina for taking care of their undergrad volunteers!! #CovidVaccine #latinasinmedicine I’m a Black woman. I’m immuno compromised. As soon as I can, I’m getting the shot. It doesn’t have any Covid in the actual vaccine. It does have the side effects of sore arm, fever, fatigue. Some people don’t have any. A lot of people take the day after off to recover from the shot. actually wearing our masks and social distancing/getting the vaccine when it’s available for us -dismantling systemic racism against BIPOC … Get the vaccine, there are plenty of famous black folks that have gotten the vaccine. Covid is killing us the most and blacks not getting the vaccine is a suicide.
Advocacy through reproach	Black and brown people are dying and have been from many different things, including this coronavirus, for a very long time. Science is real. Take the f*cking vaccine when it’s available. This is not a f*cking game you f*cking idiot. These people are horrible. Antivax people could prolong the pandemic. How many of them were privileged white people preventing working class people and people of color from getting the life saving vaccine? It’s idiocy. If you don’t vaccinate everyone, you don’t solve the pandemic. Vaccinate everyone, no matter their immigration status …
A need for a vaccine for COVID-19 and racism	Then it’s time to break racism. Like COVID, there isn’t a vaccine but it’s spread has to be stopped. As a black/Hispanic racism is real in this country. For me I TRY and rise above it and focus on what I can change and hopefully how I treat people can help those who are ignorant and judge you before they even get to know u. We need a vaccine for ignorance as well as Covid
Vaccine development and efficacy	What happened at WARP speed was that the Chinese discovered the entire genome of COVID 19 before the end of January 2020. And then they made the genetic code public. By the middle of February many labs had already come up with the basic design of the new vaccines As an optimist I see that Johnson & Johnson reported vaccine efficacy of 72% against US Covid and 57% against the South African variant This single shot vaccine was 100% effective at preventing hospitalization and death across all variants. Amazing news for humanity
Racist vaccine humor	If you’ve ever drank from an opaque plastic cup that faintly smelled of bleach and whatever the last guy was drinking at a Mexican restaurant, don’t worry about what’s in the covid vaccine. If you’ve eaten bowls from Mongolian Grill don’t worry about what’s in the covid vaccine
News updates	After nearly 10 months in the pandemic, the COVID-19 vaccine is the “beginning of the end” for a lot of Coloradans. But there are others who are more than a little hesitant. I spoke to local POC about how they view the vaccine and their responses are night and day. #9NEWS [URL] FIRST DC VACCINE: at 1:10-Lt. Jackson says she initially did not want the vaccine. But she wants to encourage black and brown people. She is sole provider for her family and father. Her longtime nurse friend has fallen very sick with COVID … [URL] Navajo Nation will receive first doses of COVID-19 vaccine early this week [URL]
Vaccine opposition (130 tweets; 12% of the sample)	
Direct Opposition	Couldn’t ever ever ever pay me to take a Chinese virus vaccine OR WEAR A MASK. IM NOT PAYIN FOR A COVID 19 VACCINE. I WILL TAKE MY CHANCES. THESE N*GGAS NOT PUTTING THAT SH*T IN MY BODY A VACCINE JUST THE VIRUS WIT SOME OTHER SH*T MIXED IN BRUH? The only ones that are ignorant are the ones that wear the stupid slave muzzles and avoid other people out of fear of something that’s in the air all the time. It’s always been there. Coronavirus by definition is the common cold. Masks are bad. Tests are bogus. Vaccines are bad.
Vaccine hesitancy	Ugh soon as Biden is elected they find a vaccine for COVID, Imma just let the white people go first The initiative to publicly vaccinate Black and Brown people against COVID in an effort to encourage other Black and Brown people to get vaccinated is cute and all … but why are they going first? There aren’t White doctors and nurses readily available orrrrrrr i want to take the COVID-19 vaccine, but I want to be educated more about it, especially being an African American man. And with what I heard about Hank Aaron, him taking the vaccine and few weeks later he died, then the y try to say he died of natural causes
Adverse reactions	… Black folks are skeptical of #COVID19 vaccine. So many have autoimmune diseases, there will likely be a higher incidence of bad reactions, if not deaths, amongst Blacks who opt for vaccination If I get covid, I have a much higher chance of not dying but didn’t the black woman who is a nurse just get paralysis on one side of her face from the vaccine and said she wouldn’t recommend it to her worst enemy? … yea no.
Conspiracy and Misinformation (161 tweets; 15% of the sample)	
Scientific misinformation	They are targeting people of color using Black Doctors to say its safe but can’t name 1 d*mn researcher. It’s redesigning DNA now COVID-19 is real but it’s no vaccine to a biochemical agent which is SARS 2.WHO changed the name to a virus to trick the masses Don’t TAKE THAT MESS The CCP bio weapon COVID-19 causes infertility. Vaccine will attack a ladies fertility because it attacks a spike protein necessary for production of a placenta. No more than regular immunity does. Biden’s master, Xi Chinese Communist Party attacked fertility. Get pregnant Now! I had a patient’s dad tell me tonight that the reason he’s heard that Hispanic people aren’t getting the covid vaccine is because they think the devil is in it. He was very interested to know my experience with it. #COVID19
Political misinformation	Introduce the micro-chip. Free covid-19 vaccines for everybody, free health care for all, introduce free illegall immigration. My coworkers tell me on a daily basis at least 2 of these things: Covid is fake Election is rigged The world is coming to an end The vaccine is being used for population control Black people aren’t oppressed Muslims are the cause of all bad things
Beliefs about immunity and protective behaviors	90% of the people I interacted with at this wedding tested positive for the corona virus. Apparently my immune system is a brick wall and based of this data I’d like to let everyone with Hispanic origins know we are immune to COVID-19!! Go out and celebrate we are immortal!! If you take the vaccine, just know how bad the side effects can be and potentially give you the effects of you having this Wuhan-Chinese Originated Virus! Remember if you eat healthy, keep immune system healthy and take Vitamins, you have nothing to worry about!
Race extermination conspiracy	Anyone Black in Amerikkka SHOULD NOT take the COVID vaccine. It’s poison and designed to eliminate people of color. If we didn’t learn from the Tuskegee Experiment then we deserve death! F*ck me once, shame on u. F*ck me twice shame on me! This a*s says exit only!!! GTFOH!!! I think Covid was designed to kill off POC …
Equity and Access (203 tweets; 18% of the sample)	
Overcoming history of medical racism	Tuskegee and COVID vaccine: The massive crime of Tuskegee is knowing there was syphilis treatment and not offering it. It would similarly be a massive crime to have a vaccine that works and not offer it. Medical racism is incontrovertibly real; helping you get a vaccine isn’t. It is one thing to blame America for the past. But if Black people choose not to take the COVID-19 vaccine, we can blame only ourselves for what happens to our community. When it comes to the #CovidVaccines, fear is not an option for African Americans
Pointing out health disparities	The mortality rate of POC with #diabetes facing COVID is terrifying. The immune compromised are forced into extreme social isolation in fear of contracting it, while potentially losing protections for our pre-ex conditions; choosing between rent, food, & insulin Seems legit: American Indians are 4x more likely to be hospitalized for COVID-19 than white people & more than 2x as likely to die. For all these reasons, past and present, Hedrick says, Indigenous people should be moved toward the front of the line to receive a vaccine. If immigration status makes someone ineligible for the vaccine it should make them ineligible to work in crowded factories that were dangerous even before covid. (And F this guy honestly, how dare he?!).
Facilitators to vaccine access	As a result of the meeting w/Dr Bruce Dart @TulsaHealthDept Director, today I participated in the Latinx COVID Outreach Committee Meeting. Vaccine application process, hospitalization admissions & how to convince Hispanics about the benefits of the vaccine, the major issues. Navajo Doctors, including my dear friend @drmichelletom, are receiving the #COVID19 vaccine. Now, the strategy needs to prioritize Indigenous, Black and Brown communities who have been hit worse by the pandemic, i.e., citizens and members of the Navajo Nation. Undocumented workers could potentially be the last to get the COVID vaccine. The virus doesn’t discriminate on status, neither should states in the distribution of vaccines. Sign @unitedwedream petition NOW and demand vaccines for ALL undocumented workers
Representation (146 tweets; 13% of the sample)	
Pride in vaccine development	APPRECIATION TWEET: COVID-19 Vaccine developed by a Black woman. First doses of the vaccine given to Black women healthcare workers on the frontlines in Louisiana and New York! Show some appreciation to a Black woman today! Dr. Fauci explains that the Covid-19 vaccine you are going to be taking, was developed by an African American Woman! That’s just the facts! Black woman magic @KizzyPhD created the COVID vaccine.
Role Models	A small group of our staff who qualified for the COVID-19 vaccine received their first doses sending the message that is safe & setting the example for others in the Latino community who have make their own decision to take it! Civil rights leaders and Black sports icons are publicly taking COVID-19 vaccines to encourage African Americans to follow their example as social media misinformation exploits Black distrust of vaccines #COVID19 #coronavirus
Politics (222 tweets; 20% of the sample)	
Role of politics in vaccines	Any politician calling for “compulsory mask wearing and social distancing,” “wait for the vaccine to end the pandemic,” and “more lock downs are necessary,” etc. has self-identified as a Khazarian Mafia Slave (KMS). @SenRonJohnson @realDonaldTrump 2.2 million U.S Citizens were expected to die from the the Communist Chinese Virus, imo President Trump’s solid leadership saved 2 million lives and produced a vaccine in 10 months that normally would have taken 5 years to make. President Trump may have a Billion lives. The Pentagon blocked members of President Joe Biden’s incoming administration from gaining access to critical information about current operations, including the troop drawdown in Afghanistan, upcoming special operations missions in Africa and COVID-19 vaccine distribution!
International politics	Malicious! Palestinians aren’t excluded from Israeli Covid vaccine rollout. - They rejected Covid cooperation w/Israel - They’re in charge of own health care under Oslo Accords - They spurned UAE’s Covid aid - They’re awaiting millions of doses of Russian vaccine. An apology? Brazilian supreme court decides all Brazilians are required to be vaccinated against COVID-19. Those who fail to prove they have been vaccinated may have their rights, such as welfare payments, public school enrolment or entry to certain places, curtailed.
Tweets not relevant (19 tweets; 2% of the sample)	

## Data Availability

Twitter data were collected using Twitter’s Application Programming Interface (API). Twitter’s API is free and open to the public.

## Appendix A. Vaccine-Related Key Word List

Vaccine
Vaccine
Vaccines
Vaccine
Vaccinated
Vaccinate
Vaccinations
Immunize
Immune
Immune
Immunize
Immunization
Imunization
Immunization
Immunizations
Immunisations
Antivax
Antivaxx
Anti-vaxxer
Anti-vaxxers
Anti-vaxer
Anti-vaxers
Anti vax
Antivaxxer
Antivaxxers
Antivaxxing
Vax
Vaxx
